# New Frontiers on the Metabolism, Bioavailability and Health Effects of Phenolic Compounds

**DOI:** 10.3390/molecules22010151

**Published:** 2017-01-17

**Authors:** Pedro Mena, Rafael Llorach

**Affiliations:** 1 The Laboratory of Phytochemicals in Physiology, Department of Food Science, University of Parma, 43125 Parma, Italy; 2 Biomarkers & Nutrimetabolomic Laboratory, Nutrition and Food Science Department, XaRTA, INSA, Campus Torribera, Pharmacy Faculty, University of Barcelona, 08028 Barcelona, Spain; rafallorach@ub.edu

Phenolic compounds, plant-derived secondary metabolites, have shown promising health features in epidemiological and human intervention studies dealing with the prevention of non-communicable diseases. (Poly)phenolic substances are metabolized and turned into bioavailable molecules that are able to impact different biological processes related to human health. The elucidation of the metabolic fate of (poly)phenolic constituents and their bioavailability is a tipping point to fully unravel the bioactive(s) responsible for the preventative effects of phenolics in the framework of cardiovascular diseases, metabolic syndrome, neurodegenerative disorders, and certain kinds of cancer. However, although much has been reported on this topic during the last few decades, every new contribution to the world of bioavailability and bioactivity of phenolic compounds usually identifies new frontiers to be explored. This Special Issue provides a series of 18 original contributions, 13 research articles and 5 reviews, dealing with some of the multiple aspects behind the metabolism and potential health properties of phenolic compounds or foods rich in this class of phytochemicals.

The accurate characterization of the phenolic composition of a plant product is key to further evaluate its biological properties. In this sense, the identification of a total of 66 (poly)phenolic compounds in a dried spearmint extract carried out by Cirlini et al. [1] represents a good example of phytochemical profiling. Once the phenolic fraction of a plant matrix has been well established, the preparation of plant foods or polyphenol extracts protecting or enhancing the phenolic content has to be considered. Fujioka et al. [2] evaluated the effect on tea composition of powdering green tea leaves with a ceramic mill in comparison to leaf steeping. They showed that this procedure allows the extraction of three times more epigallocatechin-gallate than from normal leaf tea, which in turn might also improve the in vitro inhibitory effects of green tea on the production of reactive oxygen species [2]. In this context, Reyes-Diaz et al. [3] reviewed the relevance of methyl jasmonate as an alternative to improve the phytochemical content and health properties of pre- and post-harvest fresh fruits. They concluded that the application of methyl jasmonate may enhance the production of bioactive compounds such as anthocyanins, non-coloured flavonoids, and phenolic acids [3]. Mojzer et al. [4] highlighted the importance of some extraction techniques, such as supercritical fluids, to improve or protect the phenolic composition of plant extracts. They also commented on the role that nanoformulations may have in enhancing the limited bioavailability of some polyphenols, and the potential synergistic effects of some polyphenol mixtures or their combinations with drugs [4]. Tumbas-Šaponjac et al. [5] optimised by response surface methodology (RSM) the encapsulation of beetroot pomace bioactives, as a method to valorise food by-products. In addition, they evaluated the gastrointestinal stability of the phenolic fraction by using an in vitro digestion model, enriching their work with an interesting multidisciplinary approach [5].

Several works, using different methodologies, focused on the bioavailability and metabolism of phenolics compounds. Some of these works took into account key aspects, such as the role of the gut microbiota in the biotransformation of phenolic compounds and in their inter-individual differences [6,7]. In this sense, Gaya et al. [6] evaluated the microbial metabolism of ellagitannins, isoflavones, and lignans by using fecal starters of 14 subjects, and studied the possible correlations in the metabolism of these three groups of polyphenols. Feliciano et al. [7] assessed plasma and urinary phenolic profiles after acute and daily consumption of wild blueberries in 18 healthy men. The authors quantified as many as 61 phenolic metabolites and provided novel insights in the inter-individual variability existing in their circulating levels. By using a rat model, Ma et al. [8] studied the tissue distribution and urinary excretion of gallic acid and protocatechuic acid after the intake of an aqueous extract of *Polygonum capitatum*, also identifying some methyl-conjugated metabolites in urine. Ferrazzano et al. [9] designed a chewing gum containing quercetin and evaluated its bioaccessibility in human saliva. They also assessed the antibacterial activity of the quercetin-enriched chewing gum against oral *Streptococcus mutans* after 14 days of daily consumption [9].

When studying the potential bioactivity of phenolic compounds in cells, the circulating metabolites, rather than the in planta compounds, should be applied. Spigoni et al. [10] tested the effect of some urolithins, ellagitannin-derived metabolites of colonic origin, on nitric oxide bioavailability in primary human aortic endothelial cells (HAECs). Besides single compounds, they used molecular mixtures, an approach that should always be pursued. They also studied the peripheral metabolism of urolithins in cell media, another interesting aspect to be fully considered in further in vitro studies [10]. Nevertheless, the use of real circulating metabolites is not required when phenolic compounds are topically administered to, for instance, assist wound healing processes. In this regard, Yang et al. [11] thoroughly evaluated the efficacy of gallic acid to promote wound healing in normal and hyperglucidic conditions. Similarly, Iriondo-DeHond et al. [12] assessed the antiaging properties of a coffee silverskin extract on human keratinocytes. To support the antiaging properties of the extract, they also used, as an in vivo model, *Caenorhabditis elegans* treated with ultraviolet radiation C. This cell/animal study might contribute to increase the coffee sector’s sustainability through the application of by-product extracts to preserve skin health. On the other hand, Calabriso et al. [13] studied in detail the protective role of red grape skin polyphenols in matrix metalloproteinase-2 and -9 activity and their expression in cell models of vascular inflammation. Some remarkable methodological aspects of this work are represented by the use of phenolic extracts from different cultivars and the study of the specificity of different classes of polyphenols to exert their preventive actions [13].

The work published by Chis et al. [14] showed an interesting methodology. In particular, they showed the in vivo synergistic effect of quercetin and moderate exercise on improving aortic tissue injuries and oxidative stress markers in diabetic rats. In a placebo-controlled human intervention study, Villaño et al. [15] investigated the effect of a polyphenol-rich drink on iron metabolism in 16 elite triathletes following a long-term training intervention of 120 days. They measured plasma concentrations of the hormone hepcidin, implicated in iron metabolism, and observed the lack of interference in iron absorption of phenolic compounds from an aronia-citrus juice [15]. The insights of this study can help to design, with an evidence based approach, specialised polyphenol-rich sport foods targeted to achieve a well-balanced diet in triathletes.

Most of the experimental works published within this Special Issue accounted for the bioactivity of phenolic compounds in multiple contexts. Some authors also contributed to this topic with literature reviews highlighting the importance of phenolic compounds in the prevention of some (patho)physiological conditions. Moga et al. [16] reviewed the role of polyphenols in the prevention and treatment of cervical cancer, an important cause of death of women worldwide. They showed the great potential of some polyphenols in the prevention of this cancer, but they also indicated the resistance of cervical tumour cells to chemo- and radiotherapy in the presence of some polyphenols, with an obvious negative aftermath on the efficacy of the therapy [16]. Parkison and Cicerale [17] reviewed the health benefits attributed to the phenolic fraction of virgin olive oil. They highlighted the preventive effects of oleurepein, hydroxytyrosol, and oleocanthal in cell and animal models, and commented on the need for human intervention studies to confirm the prospects of olive oil phenolics on chronic disease prevention. Finally, Martini et al. [18] summarized the main findings on the effect of coffee consumption on the modulation of oxidative stress biomarkers. They considered 26 acute and chronic human intervention studies and evaluated the protection of coffee consumption against lipid, protein, and DNA oxidative damage, as well as on antioxidant enzymes. Their findings and critical comments on literature limitations that must be overcome in future studies are of notable interest.

In summary, many different aspects have been considered within this Special Issue. However, there is still work to be done. We hope these works will inspire the readers to explore the frontiers of metabolism, bioavailability, and the health effects of phenolic compounds…and, perhaps, will help them in identifying new frontiers to be explored.
